# A medical consultation simulation in a preclinical biochemistry seminar: Does training in a high-fidelity simulation practice provide an advantage over a simulation in a traditional seminar room?

**DOI:** 10.3205/zma001845

**Published:** 2026-04-15

**Authors:** Leonard K. Saitta, Melissa Meral, Tobias M. Böckers, Achim Schneider, Susanne J. Kühl

**Affiliations:** 1 University of Ulm, Faculty of Medicine, Institute for Biochemistry and Molecular Biology, Ulm, Germany; 2 University of Ulm, Faculty of Medicine, Institute of Anatomy and Cell Biology, Ulm, Germany; 3 University of Ulm, Faculty of Medicine, Dean of Studies, Department of Studies and Teaching, Ulm, Germany

**Keywords:** simulation training, medical consultation, simulation subjects, high-fidelity simulation training

## Abstract

**Objective:**

: The ability to talk to patients is an important medical competence. Therefore, new teaching methods need to be developed and their usefulness and effectiveness studied. The objective of the study was to determine whether there are differences in the acquisition of communication skills or student evaluation depending on whether the simulated conversation is carried out in a high-fidelity simulation practice (“To Train U”, TTU) or in a traditional seminar room (SR).

**Methods:**

The study was carried out in the context of the biochemistry seminar titled “from genes to proteins”, which is part of the preclinical study portion of the Ulm School of Human Medicine. In addition to biochemical fields of study, it also includes a simulated consultation to train medical communication. One group carried out the simulated consultation in a seminar room (SR group, *n*=91), the other group in a high-fidelity simulation practice (TTU group; *n*=131). Both groups completed a communication competency test before and after the simulation, which was used to test Bloom’s level II-IV. An online evaluation was completed after the simulation.

**Results:**

Both groups showed significant increases in their overall communication competence scores. The TTU group improved significantly with regard to Bloom levels II and IV, the SR group only with regard to Level II. The TTU group reported higher satisfaction levels and perceived the simulation to be more realistic and conducive to learning than the SR group.

**Conclusion:**

High-fidelity simulation practices provide an innovative learning environment that enhances the student skills, motivation and satisfaction. Future studies should examine whether training in such settings may have lasting effects on the medical practice.

## Zusammenfassung

**Zielsetzung:**

Die ärztliche Gesprächsführung ist eine zentrale medizinische Kompetenz. Daher müssen neue Lehr-Methoden entwickelt und deren Nutzen und Effektivität untersucht werden. Ziel der Studie war es, zu prüfen, ob es Unterschiede im Erwerb der Kommunikationskompetenz oder der Studierendenevaluation gibt, abhängig davon, ob das Simulationsgespräch in einer High-Fidelity-Simulationspraxis („To Train U“, TTU) oder im traditionellen Seminarraum (SR) durchgeführt wird.

**Methodik:**

Die Untersuchung fand im Biochemie-Seminar „Vom Gen zum Protein“ des vorklinischen Studienabschnitts der Humanmedizin Ulm statt, das neben biochemischen Inhalten auch ein Simulationsgespräch zur Übung ärztlicher Kommunikation beinhaltet. Eine Gruppe führte das Simulationsgespräch in einem Seminarraum (SR-Gruppe, *n*=91), die andere Gruppe in einer High-Fidelity-Simulationspraxis (TTU-Gruppe; *n*=131) durch. Beide Gruppen bearbeiteten vor und nach der Simulation einen Test zur Kommunikationskompetenz, anhand dessen die Bloom’schen Level II-IV geprüft wurden. Nach der Simulation erfolgte eine Online-Evaluation.

**Ergebnisse:**

Beide Gruppen zeigten signifikante Steigerungen der Gesamtpunktzahl zur Kommunikationskompetenz. Die TTU-Gruppe verbesserte sich signifikant auf Bloom-Level II und IV, die SR-Gruppe nur auf Level II. Die TTU-Gruppe verzeichnete eine höhere Zufriedenheit und empfand die Simulation realitätsnäher und lernförderlicher als die SR-Gruppe.

**Schlussfolgerung:**

High-Fidelity-Simulationspraxen bieten eine innovative Lernumgebung, welche die Kompetenzen der Studierenden fördern, sowie Motivation und Zufriedenheit steigern. Künftige Studien sollten prüfen, ob das Training in solchen Einrichtungen nachhaltige Effekte auf die ärztliche Praxis hat.

## 1. Introduction

### 1.1. Background information about the study 

An effective physician/patient relationship can positively influence the success of the treatment [1], [2]. Empathic communication, including verbal and non-verbal aspects such as tone of voice, body language and facial expressions, contributes significantly to this success [3]. Communication skills training is an integral part of medical studies, since it is crucial that students learn how to convey complex content in a way that laypersons can understand. Since simulations are an effective training method [4], medical faculties have already integrated role-playing scenarios with simulated persons into their courses to help students hone their communication skills. Simulation centers with appropriate consultation rooms offer not only a safe but also a realistic learning environment [5], [6]. However, their benefits, particularly in the preclinical study phase, have not sufficiently been studied yet [4].

### 1.2. Simulation centers as innovative places of learning in medicine 

Simulations are experienced on three levels: physical, conceptual and emotional. The physical level includes aspects such as the medical equipment and the rooms. The conceptual level refers to theoretical, logical relationships, the emotional level of emotions [7]. The interplay of these levels leads to immersive learning situations, especially in high-fidelity simulation rooms [8]. It involves the use of realistic rooms that resemble a physician’s medical practice or operating room. One well-known simulation center is the “Center for Immersive and Simulation-Based Learning” at Stanford University [7]. More and more medical schools in Germany, such as the medical schools in Erlangen or Berlin, offer high-fidelity skills labs [9], [10], [11] as well. In 2021, the “To Train U” (TTU) training hospital was opened at the University of Ulm. It would be useful to scientifically examine the courses offered in this environment with regard to their effectiveness.

### 1.3. The biochemistry seminar “from genes to proteins” in Ulm

The preclinical biochemistry seminar “from genes to proteins” has been based on the “inverted classroom” concept for several years and, in addition to teaching fundamentals of biochemistry, also includes a medical consultation [12], [13], [14], [15], [16], [17]. In this context, students are asked to act as the physician and to explain the brittle bone disease (*osteogenesis imperfecta*) of their (grand)child to a family member in layperson’s terms. Initially, the simulated conversation was practiced in a traditional seminar room (with the lecturer, the students and persons participating in the simulation in the same room). 

### 1.4. Objectives of this study

The present study seeks to determine whether there are differences in terms of student competency and the evaluation results when the simulation is carried out in a traditional seminar room or in TTU simulation rooms. In addition, the study analyzes whether there are differences depending on the student's role and the location in the TTU. 

## 2. Material and methods

### 2.1. Course concept and content as well as seminar participants

**Figure 1:**
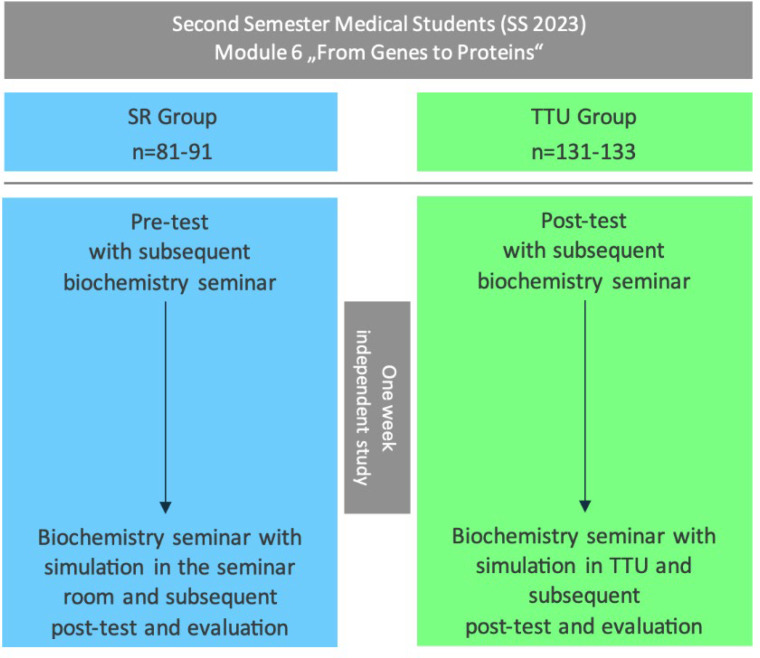
Study design Comparison of the two study groups of the integrated seminar “From Gene to Protein,” in which second-semester medical students were randomly assigned during the summer semester of 2023 as part of the regular seminar planning of the Ulm Medical Faculty. The seminar room group (SR group, control group) completed the entire seminar, including the simulation, in a conventional seminar room. The students in the TTU group (intervention group), on the other hand, carried out the simulation at the Ulm Training Hospital (TTU), while the rest of the seminar was taught in the seminar room as well. The pre-test was taken at the beginning of the first seminar session, and the post-test and evaluation at the end of the second seminar session. There was a one-week interval between the two sessions. In the SR group, 81 students completed the pre- and post-tests, and in the TTU group, 131 students. A total of 91 students from the SR group and 133 students from the TTU group participated in the evaluation. n=number of students; the number of students “n” varies depending on the number of evaluation forms or tests completed and the consent given for data processing. SS=summer semester.

The integrated “genes to proteins” seminar is offered during the 2^nd^ semester of medical studies in Ulm. It includes a medical consultation (hereinafter referred to as the “simulated session”). One student acting as the physician is asked to inform a simulated person who is a relative of a patient suffering from *osteogenesis imperfecta* about this disease. The remaining students observe the interaction and then provide feedback [12]. In the summer semester (SS) 2023, the communication training was carried out both in the traditional seminar room (SR group) and in the TTU (TTU group). The educational materials and clinical case were identical. Students were allocated to the seminar groups as part of the faculty’s seminar planning. The allocation was not randomized. For the study, six seminar groups (approx. 20 students each) were assigned to the SR group (two lecturers) and 8 seminar groups to the TTU group (one lecturer). The reason for the smaller number of groups in the SR group was to ensure that the groups were taught by only two different lecturers, thus limiting the number of different teachers and reducing potential confounding factors. *n*=81 students from the SR group and *n*=131 from the TTU group took part in the pre- and post-tests. The evaluation questionnaire was completed by *n*=91 students (SR group) and *n*=133 students (TTU group). Consent to data processing had to be given before each survey, which resulted in the different case numbers, since only data for which consent had been given was included in the analysis (see figure 1 (Fig. 1)).

#### 2.1.1. The simulation discussion: Traditional seminar room versus TTU simulation room 

##### 2.1.1.1. Simulation in the traditional seminar room

The seminar groups were each divided into three subgroups, two groups of physicians and one group of observers. One volunteer from the physicians’ groups took on the role of a physician and was briefed by the other members of the physicians’ group. For the simulated session, the physician and the patient’s family member sat at the same table. The students from the observer group observed the simulated session from the back in the same room without any technical observation elements. After the simulated session, the students received structured feedback in a group discussion, which was led by the respective lecturers [12], [13], [14], [15], [16], [17].

##### 2.1.1.2. Simulation in the TTU

The TTU groups were also divided into two groups of physicians and one group of observers per group. The physician was briefed by the respective physician groups in the same way as for the SR group. The TTU group conducted the simulated session in a high-fidelity simulated medical practice with cameras and microphones, while the other students observed the simulation from two separate rooms: an adjacent room with a view of the simulated medical practice through a one-way mirror (approx. 7 students) and an adjoining room with a livestream transmission (approx. 12 students). Just as for the SR group, structured feedback was provided during a group discussion in a conventional seminar room.

### 2.2. Competence acquisition measurement 

To objectively measure the acquisition of competence, a written test was developed in which the students had to analyze a dialogue between a physician and a relative and answer open-ended questions (see attachment 1 ). The students were able to achieve different levels of competence based on Bloom’s taxonomy [18], [19]. Successful or awkward word choices were to be marked (level II) and provided with a justification (level IV). Suggestions for improvement were to be drafted as well (level III). The pre-test focused on the starting of a conversation, the post-test on the conclusion. The requirements for both tests were identical. The completion time was 20 minutes. In total, the following points (P) were achieved in both tests:

- Total: 40 points
- Bloom level II: 14 points
- Bloom level III: 12 points
- Bloom level IV: 14 points

The tests were developed by S.J.K. and M.M., tested as a pilot during the biochemistry seminar in the summer semester 2020 with *n*=160 students, then optimized and subsequently subjected to a feedback loop by seven members of the institute. 

### 2.3. Online questionnaire

#### 2.3.1. Demographic data

The age, gender and previous education (medical training or studies) were requested [15].

#### 2.3.2. Evaluation 

The questionnaire contained 23 statements with a Likert-like scale from 1 (*“strongly disagree”*) to 6 (*“strongly agree”*). Statements 1-2 related to the TTU and statements 3-6 pertained to the individual’s motivation. Statements 7-10 dealt with the perception of the simulated session, but statement 10 could only be answered by the physician (emotional level). Statements 11-18 related to the simulated session and the feedback discussion (conceptual level). Statements 19-23 dealt with the rooms (physical level). A school grade of 1 (very good) to 6 (unsatisfactory) could be assigned. Praise, criticism and suggestions for improvement could be expressed. The majority of the statements were tested and published in previous studies [15]. The questionnaire was checked by five members of the institute before the start of the study to ensure its face validity (see attachment 2 ).

### 2.4. Study procedure and data collection

Students in both groups completed the pre-test during the first seminar session. One week of self-study took place between sessions 1 and 2. The post-test included an online evaluation (Unipark software by Tivian XI GmbH; see attachment 3 ) and was completed at the end of the second session (see figure 1 (Fig. 1)). 

### 2.5. Statistical analyses

According to the Kolmogorov-Smirnov test, the data was not normally distributed, which is why the Wilcoxon signed-rank test was used for the pre- and post-test comparison within the groups, and the *Mann-Whitney* U-test for the comparison between the groups. A *p-value* <0.05 was considered significant. As a measure for the effect size, r was calculated using the standardized *z-value (r=z/root(N))* [20]. The comparison of the categorical variables gender and prior education was carried out with a chi-square test. *IBM SPSS Statistics Version 29* for MacOS was used for the data analysis. 

### 2.6. Ethics

According to the Ethics Committee of the Ulm Medical Faculty, no ethics application was necessary. Participation in the study was unpaid, voluntary and anonymous and consent was given for data processing.

## 3. Results

### 3.1. Demographics of the study groups 

The analysis of the gender, age and previous qualifications revealed no significant differences between the two study groups (see table 1 (Tab. 1)).

### 3.2. Examination of the competence levels of the study groups before and after the simulated session

The comparison of the pre- and post-test results showed that both the SR group (*n*=81, *p*<0.001, *r*=0.44, *Md*_pre_=13, *Md*_post_=16) and the TTU group (*n*=131, *p*<0.001, *r*=0.55, *Md*_pre_=11, *Md*_post_=15) achieved a significant total score increase. 

**Figure 2:**
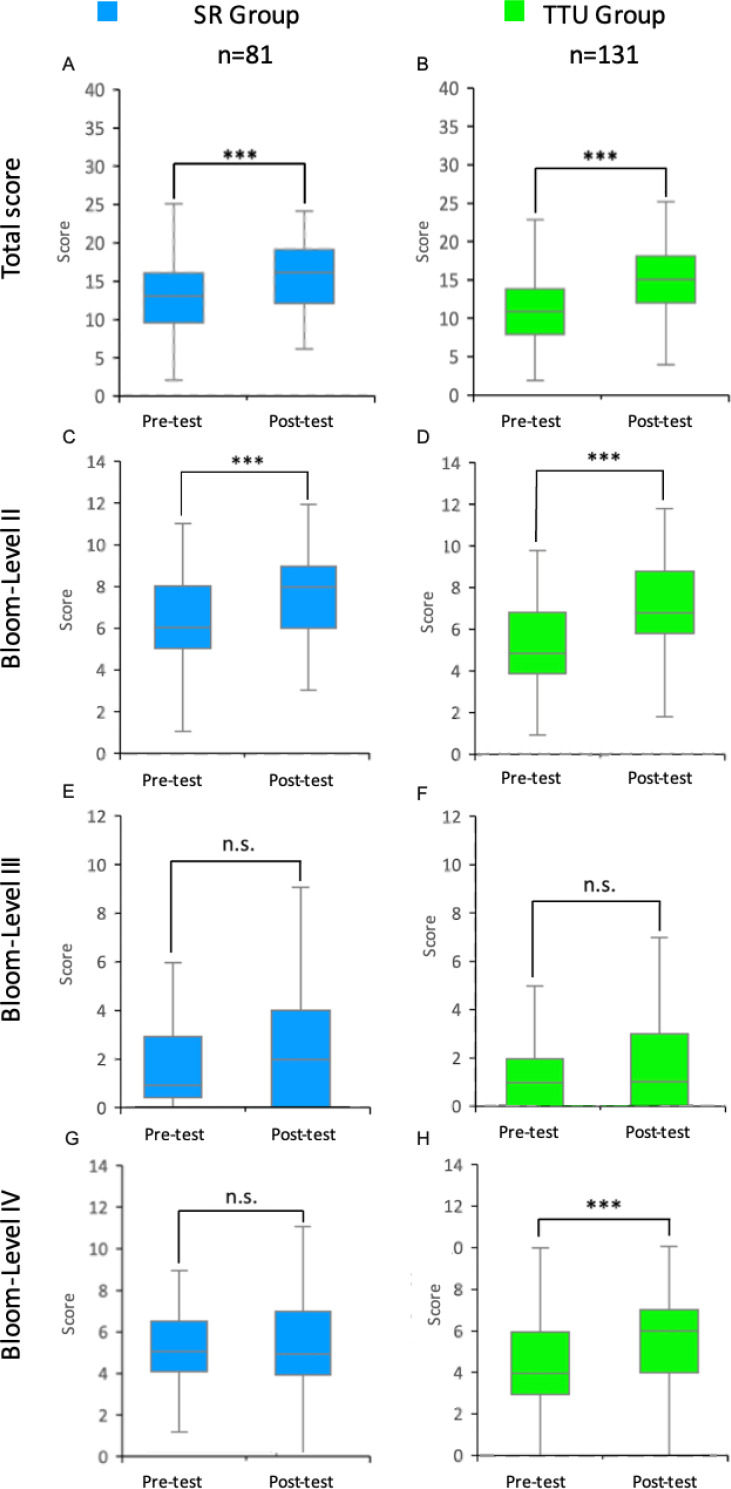
Pre- and post-test results: Differences in communication skills between the SR- and TTU-group Results of the pre- and post-tests of students in the integrated seminar “from genes to proteins” in the 2023 summer semester for the SR and TTU groups (control and intervention groups) are shown as box plots with medians. The whiskers represent the largest and smallest values achieved within the respective group. The box shows the range between the 25^th^ and 75^th^ percentiles (IQR). The median is represented as a line in the box. A total of up to 40 points could be achieved in both the pre- and post-tests. These are composed of the points for competence levels II and IV (maximum 14 points each) and competence level III (maximum 12 points). A-B: Total points scored by students in the pre- and post-tests of the SR and TTU groups. C-D: Points from the pre- and post-tests of the SR and TTU groups at Bloom Level II. E-F: Points from the pre- and post-tests of the SR and TTU groups at Bloom Level III. G-H: Points from the pre- and post-tests of the SR and TTU groups at Bloom Level IV. IQR=interquartile range, n=number of students, SR=seminar room, TTU=training hospital To Train You. Wilcoxon signed-rank test: n.s.=not significant, ***=p<0.001.

Looking at the results within the groups differentiated by Bloom’s levels, both groups showed a significant score increase at level II (understanding) (SR group: *n*=81, *p*<0.001, *r*=0.60, *Md*_pre_=6, *Md*_post_=8; TTU group: *n*=131, *p*<0.001, *r*=0.64, *Md*_pre_=5, *Md*_post_=7). At level III (application), no significant increase was observed in either group. At level IV (analysis), only the TTU group showed a significant increase from the pre-test to the post-test (*n*=131, *p*<0.001, *r*=0.41, *Md*_pre_=4, *Md*_post_=6) (see figure 2 (Fig. 2)).

### 3.3. Evaluation of the simulated session by the students 

**Figure 3:**
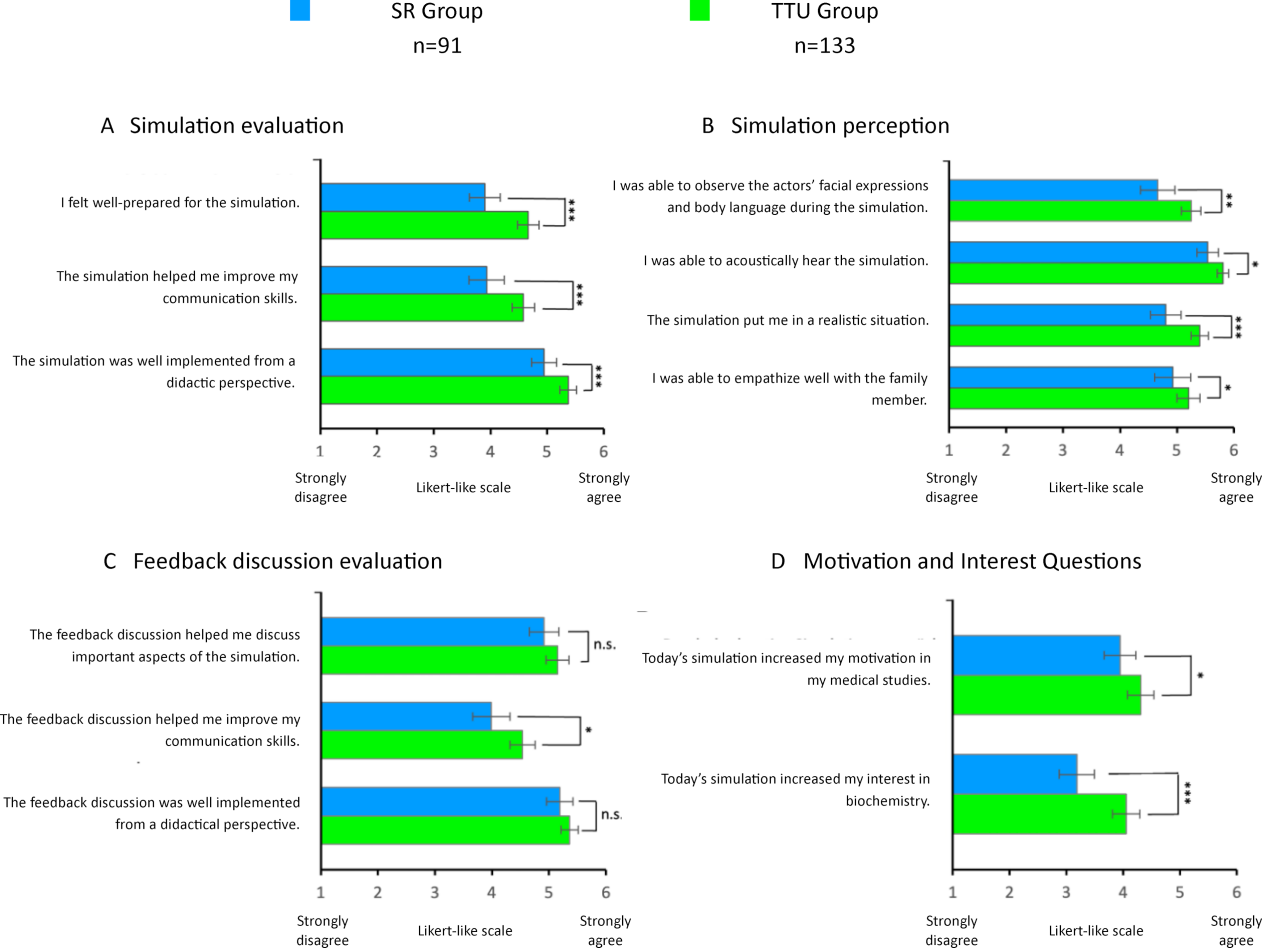
Differences in student perception and motivation between the seminar room and TTU groups Mean values from the evaluation results of students in the “from genes to proteins” seminar in the 2023 summer semester from the SR- and TTU groups (control and intervention groups). The statements were to be rated on a Likert like scale from 1 (strongly disagree) to 6 (strongly agree). The results are presented in the form of bar charts. The error bars represent the standard error. n=number of participants, SR=seminar room, training hospital *To Train You*. Mann-Whitney U test: n.s.=not significant, *=p<0.05, **=p<0.01, ***=p<0.001.

The results presented refer to selected statements from the questionnaire that showed statistically significant group differences or were particularly relevant to our research question. Not all of the statements shown in figure 3 (Fig. 3) are explained in the text. 

#### 3.3.1. Student satisfaction analysis 

The SR group rated the simulated session with an average school grade of 1.90 (*n*=90, *SD*=0.67), the TTU group with 1.50 (*n*=130, *SD*=0.60) (*p*<0.001). In general, the TTU group rated the simulated session significantly better than the SR group (“The* simulated session helped me improve my communication skills”*, SR group: *n*=91, *M*=3.93, *SD*=1.50; TTU group: *n*=133, *M*=4.58, *SD*=1.20). The TTU group found the simulated session to be more realistic than the SR group (*“The simulated session put me in a realistic situation”*, SR group: *n*=91, *M*=4.80, *SD*=1.29; TTU group: *n*=133, *M*=5.40, *SD*=0.87). The feedback discussion was also rated as more helpful by the TTU group (“The* feedback discussion helped me improve my communication skills”*, SR group: *n*=91, *M*=3.99, *SD*=1.60; TTU group: *n*=133, *M*=4.53, *SD*=1.29) (see figure 3 (Fig. 3), A-C).

#### 3.3.2. Analysis of student motivation and interest 

Before the respective simulation, both groups were comparably motivated *(“Before today's simulation, my motivation in my medical studies was high”*, SR group: *n*=91, *M*=4.78, *SD*=1.08; TTU group: *n*=133, *M*=4.9, *SD*=1.09) and interested (“Before* today's simulation, my interest in biochemistry was high”,* SR group: *n*=91, *M*=3.36, *SD*=1.49; TTU group: *n*=133, *M*=3.29, *SD*=1.31). 

After the simulation, the TTU group felt more motivation in their medical studies than the SR group (*“Today’s simulation increased my motivation in my medical studies”,* SR group: *n*=91, *M*=3.95, *SD*=1.34; TTU group: *n*=133, *M*=4.31, *SD*=1.34). Interest in biochemistry also increased significantly more in the TTU group than in the SR group (*“Today’s simulation increased my interest in biochemistry”*, SR group: *n*=91, *M*=3.19, *SD*=1.47; TTU group: *n*=133, *M*=4.05, *SD*=1.39) (see figure 3 (Fig. 3), D).

### 3.4. Detailed analysis of the TTU group

Within the TTU group, the subgroups were compared both based on their role (physician or observer) and based on the type of observation (one-way mirror or streaming room).

#### 3.4.1. Demographics of the TTU subgroups

There were no demographic differences between the subgroups (see table 2 (Tab. 2)).

#### 3.4.2. Examination of the competence levels of the TTU subgroups before and after the simulation

In the overall evaluation, there was no significant point increase for the students in the role of the physician (*n*=8, *p*>0.05, *r*=0.53, *Md*_pre_=14, *Md*_post_=16). In contrast, the students behind the one-way mirror (*n*=40, *p*<0.001, *r*=0.57, *Md*_pre_=11, *Md*_post_=15) and those in the streaming room (*n*=58, *p*<0.001, *r*=0.56, *Md*_pre_=11, *Md*_post_=15) recorded a significant increase in points. 

**Figure 4:**
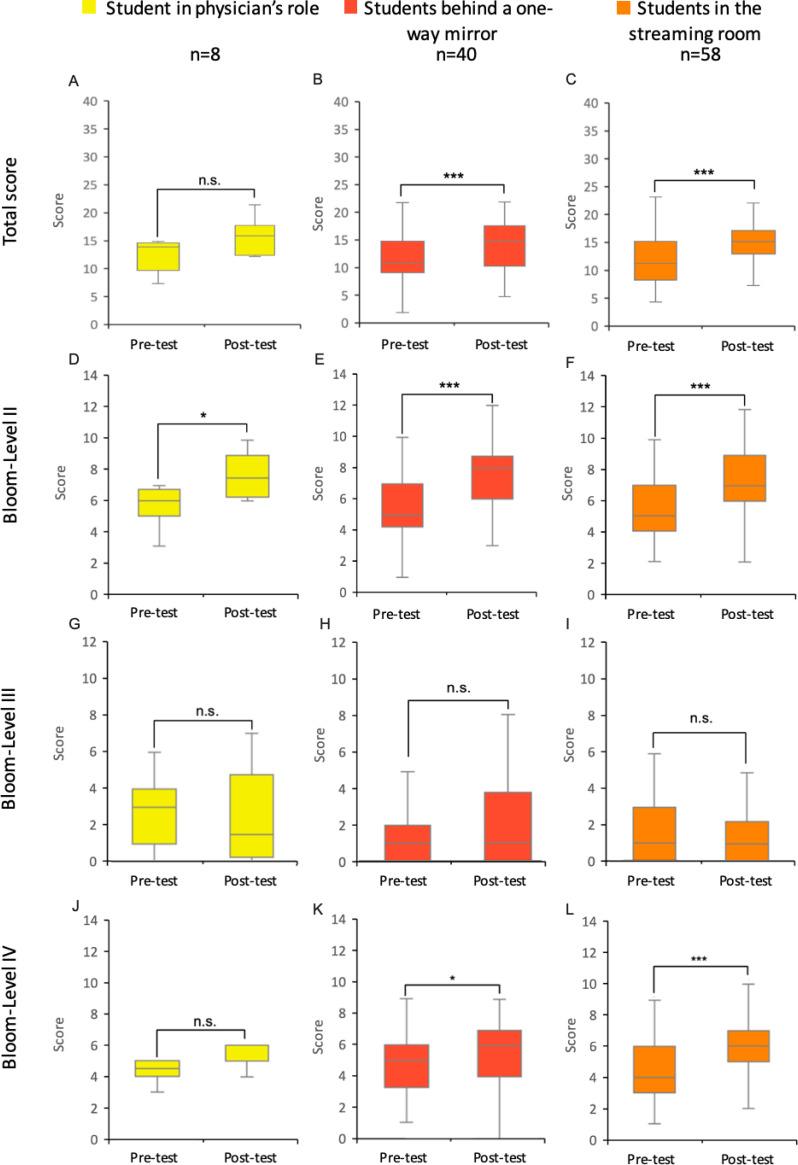
Pre- and post-test results: Differences in communication skills within the TTU subgroups Test results of students in the 2023 summer semester for the subgroups of the TTU group (intervention group). The figure shows the results of the pre- and post-tests of both groups as box plots. The whiskers represent the largest and smallest values achieved within each group. The box shows the range between the 25^th^ and 75^th^ percentiles (IQR). The median is represented as a line in the box. A total of up to 40 points could be achieved in both the pre- and post-tests. These consist of the points for competence levels II and IV (maximum 14 points each) and competence level III (maximum 12 points). A-C: Total points from the pre- and post-tests of the respective groups. D-F: Points per TTU subgroup at Bloom Level II. G-I: Points per TTU subgroup at Bloom Level III. J-L: Points per TTU subgroup at Bloom Level IV. IQR=interquartile range, n=number of students, SR=seminar room, training hospital *To Train You*. Wilcoxon signed-rank test: n.s.=not significant, *=p<0.05, **=p<0.01, ***=p<0.001.

At Bloom level II, all subgroups showed a significantly increased score between the pre- and the post-test (physician role: *n*=8, *p*<0.05, *r*=0.79, *Md*_pre_=6, *Md*_post_=7.5; behind the one-way mirror: *n*=40, *p*<0.001, *r*=0.62, *Md*_pre_=5, *Md*_post_=8; in the streaming room: *n*=58, *p*<0.001, *r*=0.59, *Md*_pre_=5, *Md*_post_=7). At level IV, both the students behind the one-way mirror (*n*=40,* p*<0.05, *r*=0.36, *Md*_pre_=5, *Md*_post_=6) and those in the streaming room (*n*=58, *p*<.001, *r*=0.44, *Md*_pre_=4, *Md*_post_=6) recorded a significant increase (see figure 4 (Fig. 4)).

#### 3.4.3. Evaluation of the simulation by TTU Subgroups

With regard to the motivation in medical studies, there was no significant difference between the subgroups before the interview *(“Before today’s simulation, my motivation in my medical studies was high”*, physician’s role: *n*=8, *M*=5.5, *SD*=0.54; behind the mirror: *n*=49, *M*=4.88, *SD*=1.11; streaming room: *n*=73, *M*=4.84, *SD*=1.12). There were significantly different levels of agreement with the interest in biochemistry before the simulation (“Before* today’s simulation, my interest in biochemistry was high”*, physician’s role: *n*=8, *M*=4.25, *SD*=1.04; behind the mirror: *n*=49, *M*=3.20, *SD*=1.21; streaming room: *n*=73, *M*=3.23, *SD*=1.36). The significance levels were reached in between the students in the physician’s role and those in the streaming room (*p*<0.05), as well in between the students in the physician’s role and those behind the mirror (*p*<0.05). 

**Figure 5:**
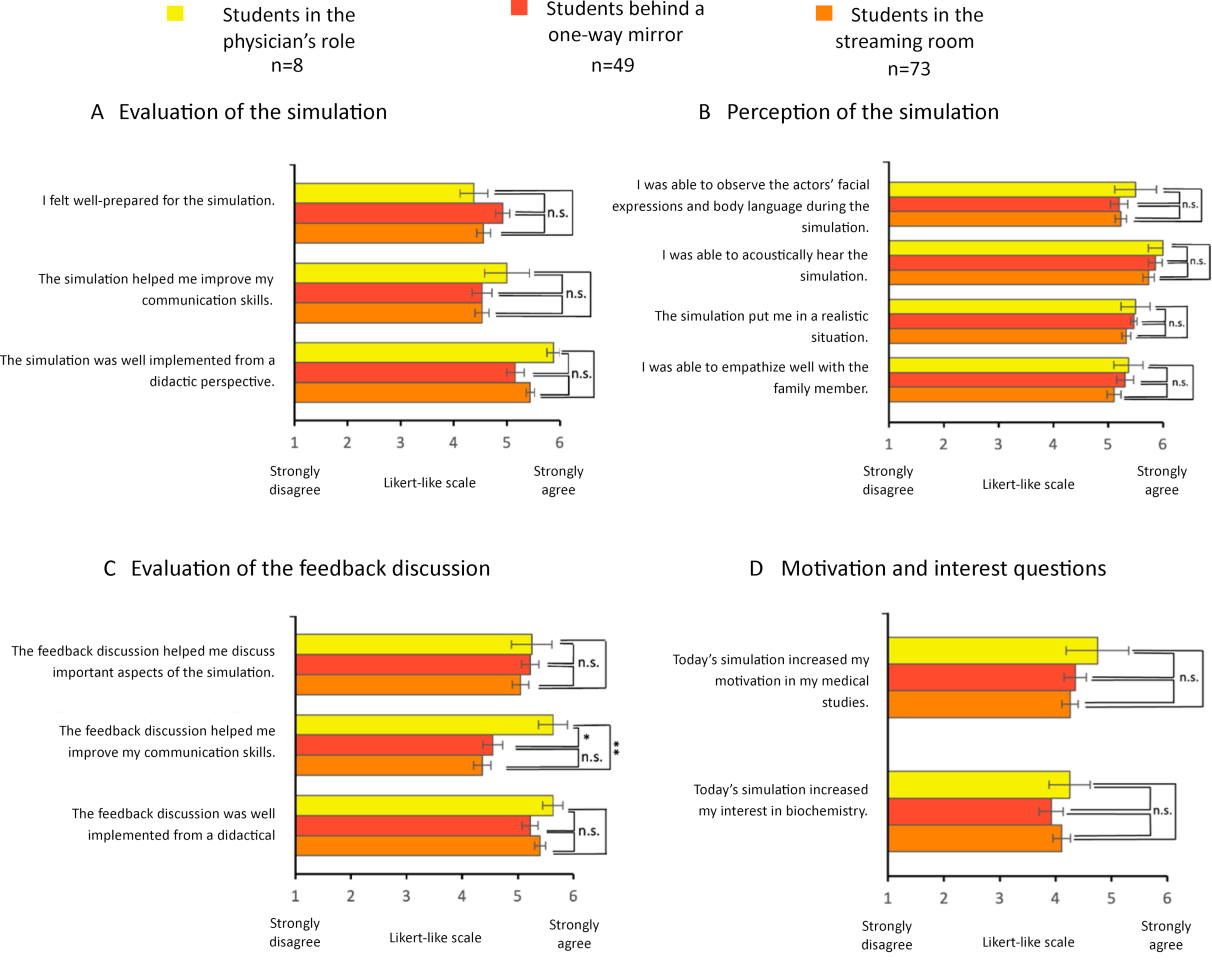
Differences in student perception and motivation within the TTU subgroups Mean values from the evaluation results of students in the “from genes to proteins” seminar in the 2023 summer semester (Intervention group) divided by roles. The statements were rated on a Likert-like scale from 1 (completely disagree) to 6 (completely agree). The results are presented as bar charts. The error bars represent the standard error. n=number of participants. Mann-Whitney U test: n.s.=not significant, *=p<0.05, **=p< 0.01.

Students in the physician’s role gave higher approval ratings for the feedback discussion *(“The feedback discussion helped me improve my communication skills”,* physician’s role: *n*=8, *M*=5.63, *SD*=0.74; behind the mirror: *n*=49, *M*=4.55, *SD*=1.26; streaming room: *n*=73, *M*=4.36, *SD*=1.32 (see figure 5 (Fig. 5)).

## 4. Discussion

The study shows that a simulation in high-fidelity learning rooms in the TTU is superior to a simulation in a conventional seminar room in terms of skills acquisition and evaluation results.

### 4.1. TTU group shows a higher level of competence and higher approval ratings in the post-simulation evaluation 

Students in the TTU group not only evaluated the simulation more positively but also achieved significantly higher scores on Bloom level IV. The immersive learning environment in the TTU thus not only promotes motivation and satisfaction but also stimulates clinical thinking in a more targeted manner. This is in line with other studies that show that realistic scenarios promote medical communication skills [21], [22]. The more positive perception of the interview preparation by the TTU group can possibly be explained by the realistic environment. This could be due to the fact that the different levels of the “physical, conceptual and emotional” reality of the simulation were addressed in more detail in the TTU group than in the SR group.

### 4.2. Differences in skills acquisition and evaluation in TTU subgroups 

Within the TTU group, the students in the role of the physician achieved a significantly higher score in the post-test at Bloom level II. The observing students, on the other hand, scored higher at levels II and IV. The observation elements used (e.g. one-way mirror, video transmission) could contribute to highlighting and reflecting communicative subtleties. This result underlines the relevance of *vicarious learning* in simulations. Several studies have shown that the pure observation of a medical conversation can also lead to an increase in learning that is comparable to active participation, especially if structured reflection processes are integrated [23], [24], [25]. As the number of participants (*n*=8) for the physician role was small, the results should be interpreted with caution. Individual outliers or differences in the group composition could influence the results. The trends presented should therefore be regarded as speculative and should be investigated further in future studies. 

### 4.3. Limitations 

All TTU groups were taught by one lecturer (S.J.K.), the SR groups by two other lecturers from the institute. However, all lecturers involved had comparable experience in teaching the seminar. 

Furthermore, for personnel reasons, the tests were evaluated by only one person (L.K.S.) and were not blinded. In order to check the reliability of the test results, some blinded samples were also evaluated by M.M. The entire test evaluation was carried out in intensive exchange with all authors. In order to minimize the practice effect of taking the test twice, the content of the two tests was different. The post-test was also completed immediately at the end of the teaching unit, meaning that it is not possible to make any statements about a sustainable skills acquisition.

Another potential influence is the Hawthorne effect, according to which students change their natural behavior under observation and evaluate it accordingly [26]. To put this into perspective, the SR group (control) was included [27]. 

In addition, no differentiation by role was made in the SR group, so that effects that are comparable to those in the TTU subgroups were not studied. 

## 5. Conclusion and outlook

Both groups showed an improvement in their medical discussion skills after the simulation, with the TTU group showing greater progress, particularly at Bloom level IV. The levels of realism within the simulation appear to have been more purposefully activated in the TTU group. The extent to which this integrated impression ultimately contributed to the higher increase in competence remains an exciting question for further studies. The positive perception of the learning environment is underlined by the significantly higher agreement of the TTU group in terms of interest and motivation. Going forward, similar studies should be carried out in TTU in connection with other courses. 

## Acknowledgements

We would like to thank all students for their participation.

## Notes

### Authorship

Achim Schneider and Susanne J. Kühl share the last authorship.

### Authors’ ORCIDs

- Melissa Meral: [0009-0005-6730-9525]
- Tobias M. Böckers: [0000-0002-1486-8535]
- Achim Schneider: [0000-0002-8602-8535]
- Susanne J. Kühl: [0000-0003-3892-3671]

## Competing interests

The authors declare that they have no competing interests. 

## Supplementary Material

Test

Excerpt of the sample solution for correcting the free text tests

Evaluation

## Figures and Tables

**Table 1:**
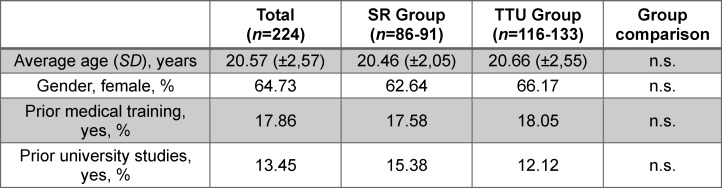
Demographic data of study participants The table shows the demographic and education-related variables of the students, presented as a mean ± standard deviation (SD) or as relative frequencies. The number of students “n” varies, because not all questions were answered by all participants. The question about previous education (training or studies) was answered with “yes” if it had a duration of at least one year (see attachment 2). n.s.: not significant

**Table 2:**
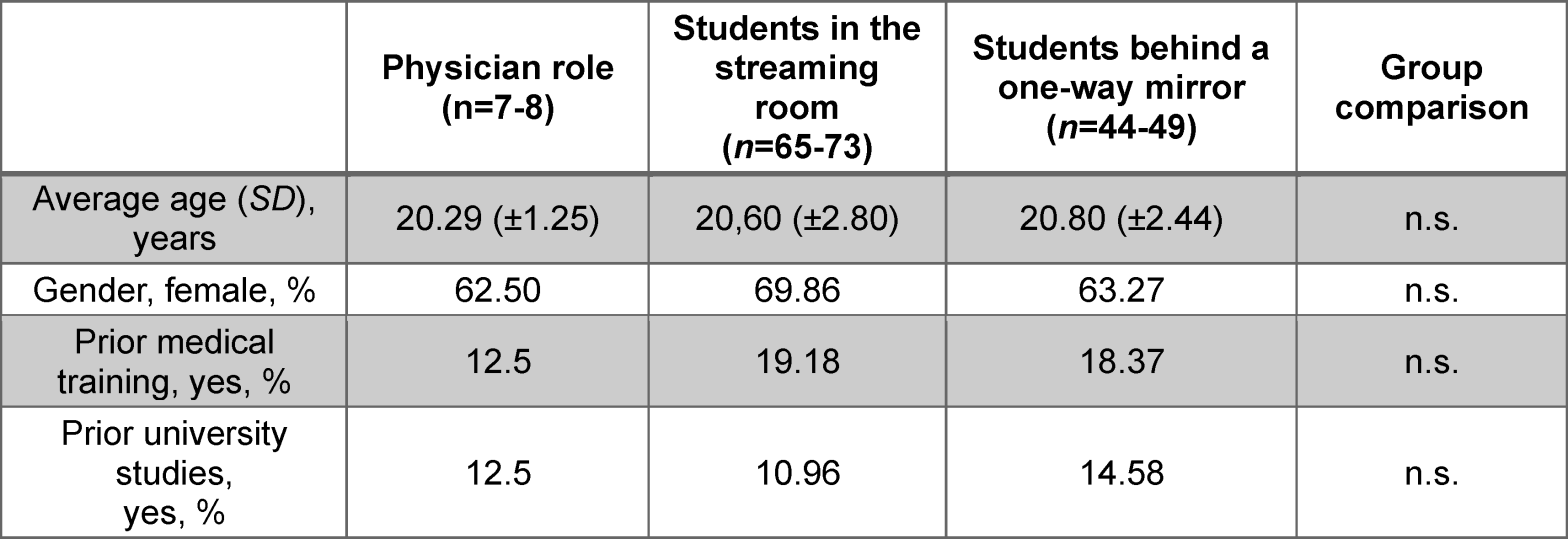
Demographic data of study participants within the TTU group The table shows the demographic and education-related variables of the subgroups within the TTU group, presented as a mean ± standard deviation (SD) or as relative frequencies. The number of students “n” varies, because not all questions were answered by all participants. The question about previous education (training or studies) was answered with “yes” if it had a duration of at least one year (see attachment 2).
