# Supplementary material for: A medical consultation simulation in a preclinical biochemistry seminar: Does training in a high-fidelity simulation practice provide an advantage over a simulation in a traditional seminar room?
Source: GMS J Med Educ. 2026 Apr 15;43(4):Doc51. doi: 10.3205/zma001845 (PMC13124465; doi:10.3205/zma001845)
Supplement: Test [file JME-43-51-s-001.pdf]

Attachment 1: Test

Physician Communication Skills

Pre-Test

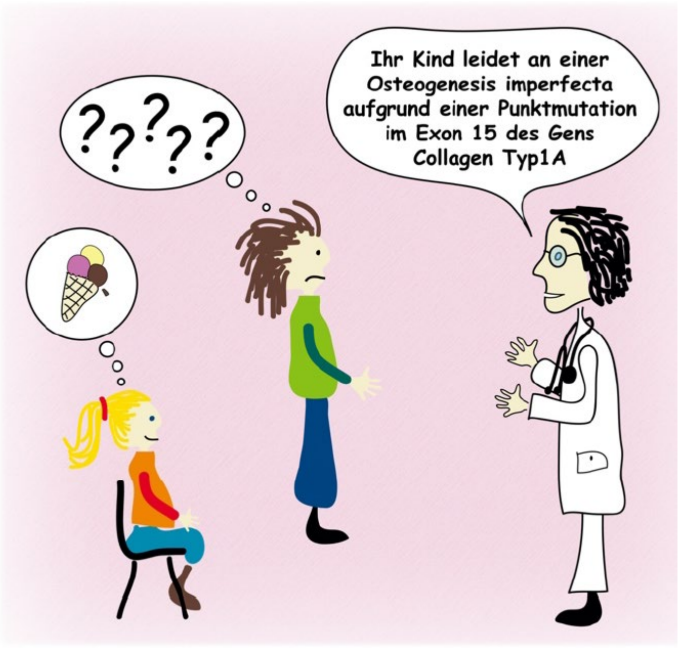

Attachment 1 to Saitta LK, Meral M, Böckers TM, Schneider A, Kühl SJ. *A medical consultation simulation in a preclinical biochemistry seminar: Does training in a high-fidelity simulation practice provide an advantage over a simulation in a traditional seminar room?* GMS J Med Educ. 2026;43(4):Doc51. DOI: 10.3205/zma001845

Assignment

Below you will find a case study and an excerpt from an informed consent discussion between you as a doctor and a mother about her son's newly diagnosed *osteogenesis imperfecta* disease. Please complete the task on your own.

In your opinion, please **mark** particularly **poorly worded text sequences** by the doctor: **provide reasons for** why you marked the text and also describe how you as a physician could respond better and what words you would choose (use the right column in the table). The **suggestions for improvement** can be either in full sentences or as bullet points, depending on the space and scope. Please pay particular attention to the patient-focused explanation of the clinical picture of *osteogenesis imperfecta* and collagen synthesis; make sure that the conversation takes into account the mother's level of knowledge and focuses on the patient.

Please also **mark** and **justify** (preferably with a different pen color) text sequences in which you think the physician **handled the conversation particularly well and / or used particularly suitable explanations** and **enter these in the right field**. Again, make sure that the clinical picture is adequately explained and that the mother is treated appropriately.

Make sure your handwriting is legible!

Case study, background about the informed consent discussion

Mrs. Mayer comes to your practice because of her 10-year-old son Sebastian. He has recently suffered several bone fractures and increasingly blue sclerae. During a previous visit to the surgical outpatient clinic for a follow-up visit, a doctor had ordered further tests (X-rays, skin biopsy, etc.) to check for *osteogenesis imperfecta*. Mrs. Mayer does not yet know anything about the specific diagnosis but was merely told that the bone fractures could have occurred in connection with a certain disease.

Mrs. Mayer is coming to your practice today to discuss the findings. As the issue is very complex, Mrs. Mayer comes to see you alone. Her son will be provided with a child-appropriate explanation of the situation at a later date. You are the physician who is to carry out the evaluation. Neither the mother nor the son have been treated by you previously. Please note that Ms. Mayer works as a retail salesclerk and therefore has little previous medical knowledge.

Attachment 1 to Saitta LK, Meral M, Böckers TM, Schneider A, Kühl SJ. *A medical consultation simulation in a preclinical biochemistry seminar: Does training in a high-fidelity simulation practice provide an advantage over a simulation in a traditional seminar room?* GMS J Med Educ. 2026;43(4):Doc51. DOI: 10.3205/zma001845

Consent to Data Processing

In order for us to be able to use your data, it is important that you consent to data processing below. We cannot use your data without your consent. You can withdraw your consent at any time by sending an email to: susanne.kuehl@uni-ulm.de

Participation is voluntary and anonymous.

I consent to data processing and data transfer for teaching research purposes by the Medical Faculty of the University of Ulm.

O Yes O No

General Personal Details

In order to ensure an anonymous allocation of the data from both surveys, please proceed as follows:

(for umlauts such as ä, ö, ü please write ae, oe, ue, twins can add a "1" for the older twin and a "2" for the younger twin in the "Place of birth" field)

... Please enter the first letter of your **place of birth** here (e.g., **U** for Ulm): ____

... Please enter the first letter of your **mother's** first name here (e.g., **M** for Monika): ____

... Please enter the first letter of your **father's** first name here (e.g., **K** for Karl): ____

... Please enter of your **mother's** birth month in numbers (two digits, e.g., **06** for June): ____

Attachment 1 to Saitta LK, Meral M, Böckers TM, Schneider A, Kühl SJ. *A medical consultation simulation in a preclinical biochemistry seminar: Does training in a high-fidelity simulation practice provide an advantage over a simulation in a traditional seminar room?* GMS J Med Educ. 2026;43(4):Doc51. DOI: 10.3205/zma001845

Conversation 1

Please mark in this column what you felt was poorly worded and what parts were well done.	***Please write down in this column what was bad or good about the conversation sequence and briefly <u>explain</u> why. Formulate <u>suggestions for improving</u> negative sequences.***
**Doctor:**<br>Hello, Mrs. Mayer, please take a seat. Do you know why you are here?	
**Ms. Mayer:**<br>Actually, not really. We know you did some tests on my son because of a possible illness. But I don’t know what this is really about, nobody told us anything.	
**Doctor:**<br>Well, as you know, your son Sebastian has had a high incidence of bone fractures in recent years in situations that are actually not very stressful.<br>As a result, the attending surgeon in the outpatient clinic recently suspected the presence of *osteogenesis imperfecta*.<br>Samples were taken from your son for diagnostic purposes and we took multiple x-rays.<br>And I have to tell you that this suspicion was unfortunately confirmed after a detailed examination of the situation.	
**Ms. Mayer:**<br>Oh, I ... that sounds terrible! What does that mean for Sebastian and for us? I've never heard of this disease before. What do we have to do? Does he have to go to hospital? And how did you even find out about all this?<br>Oh my God, my son doesn't have to die, does he?	
**Doctor:**<br>Now, let’s not get head of ourselves, Mrs. Mayer.<br>*Osteogenesis imperfecta* belongs to a group of hereditary diseases. These are characterized by a disorder in the collagen I synthesis - which causes the increased bone fragility in your son.<br>This malfunction in the bone matrix causes the bone to lose stability.	
**Ms. Mayer:**<br>I don't even know what to say about that. I don’t know much about medicine. Collagen, synthesis. Those are just words to me. What does it all mean exactly?	

Attachment 1 to Saitta LK, Meral M, Böckers TM, Schneider A, Kühl SJ. *A medical consultation simulation in a preclinical biochemistry seminar: Does training in a high-fidelity simulation practice provide an advantage over a simulation in a traditional seminar room?* GMS J Med Educ. 2026;43(4):Doc51. DOI: 10.3205/zma001845

**Doctor:**<br>I would like to try to rephrase the whole issue. What your son has is a disorder in the collagen structure - in other words, it affects the connective tissue in the body. This connective tissue is also part of the bones.<br>Your son has a mutation in a specific gene, which leads to a disruption in the formation of the type I collagen. As a result, the bones are poorly formed and abnormally elastic. This is why your son has recently and in the past repeatedly suffered from frequent fractures.<br>However, collagen is not only found in bones, but also in the cartilage of the skin, blood vessels and many other parts of our body. This is why your son has blue sclerae, for example, as you have already noticed in the past.	

[...]

Attachment 1 to Saitta LK, Meral M, Böckers TM, Schneider A, Kühl SJ. *A medical consultation simulation in a preclinical biochemistry seminar: Does training in a high-fidelity simulation practice provide an advantage over a simulation in a traditional seminar room?* GMS J Med Educ. 2026;43(4):Doc51. DOI: 10.3205/zma001845

Physician Communication Skills

Pre-Test

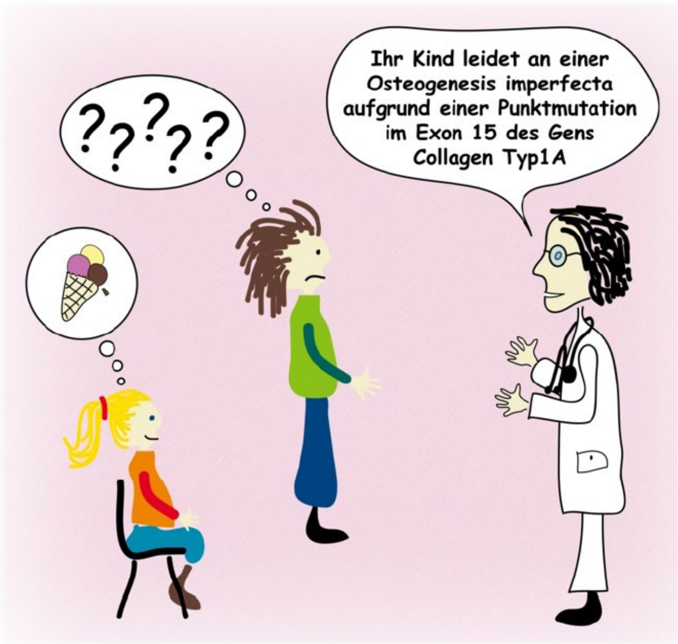

Attachment 1 to Saitta LK, Meral M, Böckers TM, Schneider A, Kühl SJ. *A medical consultation simulation in a preclinical biochemistry seminar: Does training in a high-fidelity simulation practice provide an advantage over a simulation in a traditional seminar room?* GMS J Med Educ. 2026;43(4):Doc51. DOI: 10.3205/zma001845

Assignment

Below you will see a case study and an excerpt from an informed consent discussion between you as a doctor and a mother about her son's newly diagnosed *osteogenesis imperfecta* disease. Please complete the task on your own.

In your opinion, please **mark** particularly **poorly worded text sequences** by the doctor: **provide reasons for** why you marked the text and also describe how you as a physician could respond better and what words you would choose (use the right column in the table). The **suggestions for improvement** can be either in full sentences or as bullet points, depending on the space and scope. Please pay particular attention to the patient-focused explanation of the clinical picture of *osteogenesis imperfecta* and collagen synthesis; make sure that the conversation takes into account the mother's level of knowledge and focuses on the patient.

Please also **mark** and **justify** (preferably with a different pen color) text sequences in which you think the doctor **handled the conversation particularly well and / or used particularly suitable explanations** and **also enter these in the right field**. Again, make sure that the clinical picture is adequately explained and that the mother is treated appropriately.

Make sure your handwriting is legible!

Case study, background about the informed consent discussion

Mrs. Mayer comes to your practice because of her 10-year-old son Sebastian. He has recently suffered several bone fractures and increasingly blue sclerae. During a previous visit to the surgical outpatient clinic for a follow-up visit, a doctor had ordered further tests (X-rays, skin biopsy, etc.) to check for *osteogenesis imperfecta*. Mrs. Mayer does not yet know anything about the specific diagnosis but was merely told that the bone fractures could have occurred in connection with a certain disease.

Mrs. Mayer is coming to your practice today to discuss the findings. As the issue is very complex, Mrs. Mayer comes to see you alone. Her son will be provided with a child-appropriate explanation of the situation at a later date. You are the physician who is to carry out the evaluation. Neither the mother nor the son have been treated by you previously. Please note that Ms. Mayer works as a retail salesclerk and therefore has little previous medical knowledge.

Attachment 1 to Saitta LK, Meral M, Böckers TM, Schneider A, Kühl SJ. *A medical consultation simulation in a preclinical biochemistry seminar: Does training in a high-fidelity simulation practice provide an advantage over a simulation in a traditional seminar room?* GMS J Med Educ. 2026;43(4):Doc51. DOI: 10.3205/zma001845

Consent to Data Processing

In order for us to be able to use your data, it is important that you consent to data processing below. We cannot use your data without your consent. You can withdraw your consent at any time by sending an email to: susanne.kuehl@uni-ulm.de

Participation is voluntary and anonymous.

I consent to data processing and data transfer for teaching research purposes by the Medical Faculty of the University of Ulm.

O Yes O No

To ensure an anonymous allocation of the data from both surveys, please proceed as follows:

(for umlauts such as ä, ö, ü please write ae, oe, ue, twins can add a "1" for the older twin and a "2" for the younger twin in the "Place of birth" field)

... Please enter the first letter of your **place of birth** here (e.g., **U** for Ulm): ____

... Please enter the first letter of your **mother's** first name here (e.g., **M** for Monika): ____

... Please enter the first letter of your **father's** first name here (e.g., **K** for Karl): ____

... Please enter of your **mother's** birth month in numbers (two digits, e.g., **06** for June): ____

Attachment 1 to Saitta LK, Meral M, Böckers TM, Schneider A, Kühl SJ. *A medical consultation simulation in a preclinical biochemistry seminar: Does training in a high-fidelity simulation practice provide an advantage over a simulation in a traditional seminar room?* GMS J Med Educ. 2026;43(4):Doc51. DOI: 10.3205/zma001845

Conversation 2

Please mark in this column what you felt was poorly worded and what parts were well done.	***Please write down in this column what was bad or good about the conversation sequence and briefly explain why. Formulate suggestions for improving negative sequences.***
**Ms. Mayer:**<br>So how does this collagen work? I can't quite wrap my head around it all. I always thought that bone is just bone. But the fact that the whole thing seems to be so complicated confuses me even more. What exactly is it and how does someone get this disease?	
**Doctor:**<br>What your son has is a disorder in the formation of connective tissue - specifically type I collagen.<br>The formation of this bone building block is actually strictly regulated in the body: in short, three individual so-called alpha chains are initially formed in the cell, which later combine to form a triple helix. These chains are modified by various processes such as hydroxylation and glycosylation. However, a mutation in an alpha chain of the collagen often leads to impaired triple helix formation. As a result, the bone breaks much faster than in healthy people.	
**Ms. Mayer:**<br>That all sounds pretty complicated. How could you actually see that? My son only broke a few bones. That can happen at his age, that you hurt yourself playing. I don't understand any of this.	
**Doctor:**<br>A skin biopsy of your son was already taken in the surgical outpatient clinic. We also took some x-rays. They show that your son is losing bone mass, which appears as thinning bones on the images. You can also see the lack of bone formation that we have already mentioned.<br>We then used the skin sample to carry out further tests, namely traditional DNA sequencing according to Sanger. That allowed us to separate the genetic information in the skin sample into its individual bases and then carry out further studies.<br>Here we found a change in the *COL1A1 gene.* This is a gene that is extremely important for the formation of type I collagen. The amino acid glycine is substituted by valine at this point, which means that only a small amount of functional type I collagen is formed. And this is the cause of your son's disease with all its symptoms.	

Attachment 1 to Saitta LK, Meral M, Böckers TM, Schneider A, Kühl SJ. *A medical consultation simulation in a preclinical biochemistry seminar: Does training in a high-fidelity simulation practice provide an advantage over a simulation in a traditional seminar room?* GMS J Med Educ. 2026;43(4):Doc51. DOI: 10.3205/zma001845

**Mrs. Mayer:**<br>And what do we do now?	
**Doctor:**<br>Since the whole thing has genetic causes, unfortunately there is no therapy that will correct the core of the problem. We can only treat the various symptoms. We have medication for this, but we will also work with physiotherapy. And if he breaks a bone again, it will of course be treated accordingly. We will also draw up a comprehensive prevention program to provide your son with the best possible care and protection.	
**Ms. Mayer:**<br>Phew, that's quite a lot of information. I'll have to explain it to my husband when I get home...	
**Doctor:**<br>That's about it from my side. It's best to let it all sink in first and take your time to talk about it at home. I would suggest that you make another appointment at the registration desk for next week. Then we can discuss everything else.<br>I have to get going, take care and see you next week!	

Evaluation of the Physician's Communication Skill Tests

We would like to ask you to take part in a short evaluation of the seminar. The survey will take about three minutes. Participation is voluntary and anonymous.

Please scan the QR code shown here with your device or enter the link in your browser.

https://ww2.unipark.de/uc/ttu/studie/

Attachment 1 to Saitta LK, Meral M, Böckers TM, Schneider A, Kühl SJ. *A medical consultation simulation in a preclinical biochemistry seminar: Does training in a high-fidelity simulation practice provide an advantage over a simulation in a traditional seminar room?* GMS J Med Educ. 2026;43(4):Doc51. DOI: 10.3205/zma001845
