# Supplementary material for: A medical consultation simulation in a preclinical biochemistry seminar: Does training in a high-fidelity simulation practice provide an advantage over a simulation in a traditional seminar room?
Source: GMS J Med Educ. 2026 Apr 15;43(4):Doc51. doi: 10.3205/zma001845 (PMC13124465; doi:10.3205/zma001845)
Supplement: Excerpt of the sample solution for correcting the free text tests [file JME-43-51-s-002.pdf]

Attachment 2: Excerpt of the sample solution for correcting the free text tests

**L E G E N D**	
red = poorly done<br>blue = well done<br><br>*(Level 1: basic requirement for all subsequent levels)*<br><br>Level 2: The corresponding passages are underlined (without explanation/alternative)<br>Level 3: Alternative answer or different choice of words (without explanation)<br>Level 4: Reason why good/bad (without alternative)<br><br>*(Level 5 + Level 6: not achievable in our tests)*	*No extra point*<br><br>1 point<br>1 point<br>1 point

	**PRE-TEST**	**POST-TEST**
**Maximum achievable points for level 2 (comprehension):**	14	14
**Maximum achievable points for level 3 (application):**	12	12
**Maximum achievable points for level 4 (analysis):**	14	14
	**40**	**40**

Attachment 2 to Saitta LK, Meral M, Böckers TM, Schneider A, Kühl SJ. *A medical consultation simulation in a preclinical biochemistry seminar: Does training in a high-fidelity simulation practice provide an advantage over a simulation in a traditional seminar room?* GMS J Med Educ. 2026;43(4):Doc51. DOI: 10.3205/zma001845

**Ms. Mayer:**<br>Oh, I ... that sounds terrible! What does that mean for Sebastian and for us? I've never heard of this disease before. What do we have to do? Does he have to go to hospital? And how did you even find out about all this?<br>Oh my God, my son doesn't have to die, does he?			
**Doctor:**<br>Now, let's not get ahead of ourselves, Mrs. Mayer.[5]<br><br><br>*Osteogenesis imperfecta*[6]belongs to a group of hereditary diseases. These are characterized by a disorder in the collagen I synthesis[7]- which causes the increased bone fragility in your son.[8] This malfunction in the bone matrix causes the bone to lose stability.	[5]Not enough empathy/understanding<br><br>Suggestion: "Mrs. Mayer, I understand that the situation is very upsetting for you at the moment. If it's okay with you, I would like to explain to you what this illness is all about and what causes it. That way you can better understand what is important now."<br><br>[6]Using a technical term without explaining it<br><br>Suggestion: "Brittle bone disease..."<br><br>([7]) Using a technical term without explaining it<br><br>Suggestion: "Disorder in the formation of this particular component in the bone"<br><br>[8]Complicated wording<br><br>Suggestion: "Collagen is like a braid with three strands that provides the bone with stability and support. If one of the strands is not properly structured, the entire braid is loose and the bone becomes brittle, and this is what happens with brittle bone disease."	1 point<br>1 point<br>1 point<br><br><br>1 point<br>1 point<br>1 point<br><br>1 point<br>1 point<br>1 point<br><br><br>1 point<br>1 point<br>1 point	Level 2-4<br><br><br><br><br>Level 2-4<br><br><br><br>Level 2-4<br><br><br><br><br>Level 2-4

Attachment 2 to Saitta LK, Meral M, Böckers TM, Schneider A, Kühl SJ. *A medical consultation simulation in a preclinical biochemistry seminar: Does training in a high-fidelity simulation practice provide an advantage over a simulation in a traditional seminar room?* GMS J Med Educ. 2026;43(4):Doc51. DOI: 10.3205/zma001845
