# Supplementary material for: A medical consultation simulation in a preclinical biochemistry seminar: Does training in a high-fidelity simulation practice provide an advantage over a simulation in a traditional seminar room?
Source: GMS J Med Educ. 2026 Apr 15;43(4):Doc51. doi: 10.3205/zma001845 (PMC13124465; doi:10.3205/zma001845)
Supplement: Evaluation [file JME-43-51-s-003.pdf]

Attachment 3: Evaluation

Dear students,

I would like to invite you to complete the following questionnaire as part of my doctoral thesis. Your data will be treated confidentially. The evaluation and publication of the results will be anonymous.

Please consent to data processing so that I can use the data. You can withdraw your consent at any time by sending an email to: susanne.kuehl@uni-ulm.de.

You would be very supportive if you complete the questions conscientiously. Please do NOT look for the answers on the Internet while filling out the form.

Thank you for your participation!

General information

I consent to data processing and data transfer for teaching research purposes by the Medical Faculty of the University of Ulm.

☐ yes ☐ no

In order to ensure an anonymous allocation of the data from both surveys, please proceed as follows:

(For umlauts such as ä, ö, ü please write ae, oe, ue, twins can add a "1" for the older twin and a "2" for the younger twin in the "Place of birth" field)

... Please enter the first letter of your place of birth here (e.g., U for Ulm): ☐

... Please enter the first letter of your mother's first name here (e.g., M for Monika): ☐

... Please enter the first letter of your father's first name here (e.g., K for Karl): ☐

... Please enter of your mother's birth month in numbers (two digits, e.g., 06 for June): ☐

The simulation took place in the:

☐ TTU ☐ normal seminar room

Attachment 3 to Saitta LK, Meral M, Böckers TM, Schneider A, Kühl SJ. *A medical consultation simulation in a preclinical biochemistry seminar: Does training in a high-fidelity simulation practice provide an advantage over a simulation in a traditional seminar room?* GMS J Med Educ. 2026;43(4):Doc51. DOI: 10.3205/zma001845

General information

Please enter your age: ☐

I have already completed training in a medical-related field (with/without professional work experience) (total duration >1 year) [16].

☐ yes ☐ no

I have already completed a degree (prior to human medicine)? (total duration >1 year)[16]

☐ yes ☐ no

Gender

☐ male ☐ female ☐ other ☐ not specified

Which statement applies to your role in the seminar? [16] **(TTU)**

- ☐ I was a physician
- ☐ I was an observer **in the streaming room with video transmission**
- ☐ I was an observer **in the observation room behind the one-way mirror**

I was assigned to the student group with the following role [16] **(seminar room)**

- ☐ Physician
- ☐ Observer

	Strongly disagree					Strongly agree
1. The training hospital "TTU" played a role in my choice of Ulm as a study location.	☐	☐	☐	☐	☐	☐
2. The TTU meets my expectations.	☐	☐	☐	☐	☐	☐

Attachment 3 to Saitta LK, Meral M, Böckers TM, Schneider A, Kühl SJ. *A medical consultation simulation in a preclinical biochemistry seminar: Does training in a high-fidelity simulation practice provide an advantage over a simulation in a traditional seminar room?* GMS J Med Educ. 2026;43(4):Doc51. DOI: 10.3205/zma001845

Motivation questions

3. **Before** today's simulation, my motivation in my medical studies was high [16].	Strongly disagree ☐	☐	☐	☐	☐	Strongly agree ☐
4. **Before** today's simulation, my interest in biochemistry was high [16].	Strongly disagree ☐	☐	☐	☐	☐	Strongly agree ☐
5. Today's simulation increased my **motivation** in my medical studies [16].	Strongly disagree ☐	☐	☐	☐	☐	Strongly agree ☐
6. Today's simulation increased my **interest** in biochemistry [16].	Strongly disagree ☐	☐	☐	☐	☐	Strongly agree ☐

Perception questions

7. I was able to observe the actors´ facial expressions and body language during the simulation.	Strongly disagree ☐	☐	☐	☐	☐	Strongly agree ☐
8. The simulation put me in a realistic situation [16].	Strongly disagree ☐	☐	☐	☐	☐	Strongly agree ☐
9. I was able to acoustically hear the simulation.	Strongly disagree ☐	☐	☐	☐	☐	Strongly agree ☐
10. I felt like I was being observed by my fellow students during the simulation (physician role).	Strongly disagree ☐	☐	☐	☐	☐	Strongly agree ☐

Attachment 3 to Saitta LK, Meral M, Böckers TM, Schneider A, Kühl SJ. *A medical consultation simulation in a preclinical biochemistry seminar: Does training in a high-fidelity simulation practice provide an advantage over a simulation in a traditional seminar room?* GMS J Med Educ. 2026;43(4):Doc51. DOI: 10.3205/zma001845

Simulation discussion and feedback

11. I felt well-prepared for the simulation.

Strongly disagree ☐ ☐ ☐ ☐ ☐ ☐ Strongly agree

12. The simulation helped me to improve my communication skills.

Strongly disagree ☐ ☐ ☐ ☐ ☐ ☐ Strongly agree

13. The simulation was well implemented from a didactic perspective.

Strongly disagree ☐ ☐ ☐ ☐ ☐ ☐ Strongly agree

14. The feedback discussion was well implemented from a didactic perspective.

Strongly disagree ☐ ☐ ☐ ☐ ☐ ☐ Strongly agree

15. The feedback discussion helped me to discuss important aspects of the simulation. [16].

Strongly disagree ☐ ☐ ☐ ☐ ☐ ☐ Strongly agree

16. The feedback helped me improve my communication skills.

Strongly disagree ☐ ☐ ☐ ☐ ☐ ☐ Strongly agree

17. Overall, I found the seminar very conducive to learning.

Strongly disagree ☐ ☐ ☐ ☐ ☐ ☐ Strongly agree

18. I was able to empathize well with the family member

Strongly disagree ☐ ☐ ☐ ☐ ☐ ☐ Strongly agree

Questions about the premises

19. The simulation rooms were particularly conducive to learning.	Strongly disagree ☐	☐	☐	☐	☐	Strongly agree ☐
20. I think a training hospital is important for medical training.	Strongly disagree ☐	☐	☐	☐	☐	Strongly agree ☐
21. There were no technical issues during the simulation and feedback session.	Strongly disagree ☐	☐	☐	☐	☐	Strongly agree ☐
22. There would be better places than the seminar room in N25/ than the TTU for simulations of this kind.	Strongly disagree ☐	☐	☐	☐	☐	Strongly agree ☐

Overall assessment

Which room would you recommend for the simulation for future semesters?

☐ TTU with one-way mirror ☐ TTU with streaming room ☐ normal seminar room (N25)

	Strongly disagree					Strongly agree
23. I would like to see more simulation-based teaching formats for my further studies.	☐	☐	☐	☐	☐	☐

What grade (1-6) would you give the simulation with feedback round [16]?

Attachment 3 to Saitta LK, Meral M, Böckers TM, Schneider A, Kühl SJ. *A medical consultation simulation in a preclinical biochemistry seminar: Does training in a high-fidelity simulation practice provide an advantage over a simulation in a traditional seminar room?* GMS J Med Educ. 2026;43(4):Doc51. DOI: 10.3205/zma001845

Praise and critique

Below, you have the opportunity to praise, critique or provide some general comments about the simulation (incl. preparation and feedback round) [16]:

Below you have the opportunity to make specific suggestions for improving the simulation (incl. preparation and feedback round)[16]:

Reference

[16] Schneider A, Messerer DAC, Kuhn V, Horneffer A, Bugaj TJ, Nikendei C, Kühl M, Kühl SJ. Randomized controlled monocentric trial to compare the impact of using professional actors or peers for communication training in a competency-based inverted biochemistry classroom in preclinical medical education. BMJ Open. 2022;12(5):e050823-050823. DOI: 10.1136/bmjopen-2021-050823

- Questions on previous training, role allocation and questions no. 3-7, 15 and questions on praise, critique and the school grade were adopted identically, question no. 8 was adapted.
